# The Impact of COVID-19 Infection on Trauma Patients in South Korea

**DOI:** 10.3390/healthcare11233064

**Published:** 2023-11-29

**Authors:** Myungjin Jang, Mina Lee, Woosung Choi, Kangkook Choi

**Affiliations:** 1 Department of Trauma Surgery, Gachon University Gil Medical Center, Incheon 21556, Republic of Korea; saguri5919@gilhospital.com (M.J.); jwh@gilhospital.com (M.L.); 2 Department of Traumatology, Gachon University, Incheon 21565, Republic of Korea; 3 Department of Emergency Medicine, Gachon University Gil Medical Center, Incheon 21556, Republic of Korea; choiwoosung@gilhospital.com

**Keywords:** trauma, asymptomatic COVID-19, complication, mortality

## Abstract

Background: The COVID-19 pandemic has significantly affected societies worldwide, including the medical healthcare system and trauma care. This study explores the impact of COVID-19 infection on trauma patients in South Korea, a country with effective pandemic management. Methods: A retrospective cohort study of 4206 trauma patients from June 2020 to May 2022 was conducted. Patients were categorized into COVID-19-positive and COVID-19-negative groups. Various clinical parameters, complications, and mortality rates were analyzed. Results: COVID-19-positive patients exhibited higher rates of complications, such as pressure sores (8.8% vs. 2.3%, *p* < 0.001), surgical site infections (2.4% vs. 0.8%, *p* = 0.044), and pneumonia (8.8% vs. 4.1%, *p* = 0.007). There was no significant difference in mortality between COVID-19-positive and -negative groups (4% vs. 5.6%, *p* = 0.439). Factors influencing mortality included COVID-19 status, age, Glasgow Coma Scale, Injury Severity Score, and transfusion status. Conclusion: COVID-19 positivity may have adverse clinical effects on trauma patients, but the impact varies based on public health factors. Additional studies in different contexts are crucial to elucidate these complexities.

## 1. Introduction

The COVID-19 pandemic has had profound effects on societies worldwide and tremendous adverse impacts on healthcare systems, including the trauma system [1,2,3]. COVID-19 characteristically causes respiratory infections, including pneumonia, but it is known to have various effects on multiple systems with its unique pathophysiology [4,5,6]. The field of trauma has also been greatly impacted by the COVID-19 pandemic, which has been the subject of a tremendous amount of research. Most studies have focused on changes in the trauma care system due to the pandemic, such as the changing patterns of incidence, changes in management before and during the pandemic, and how to cope with the COVID-19 pandemic [7,8,9,10]. COVID-19 is primarily known for causing respiratory infections, but its impact extends beyond the respiratory system. The virus can induce a systemic inflammatory response, leading to a cytokine storm, which is characterized by the excessive release of pro-inflammatory cytokines [6,11]. Moreover, COVID-19 can induce coagulopathy, characterized by an increased risk of thrombosis [12,13]. This is particularly concerning for trauma patients, who are already at a higher risk for thrombotic complications. The interaction between COVID-19-induced coagulopathy and trauma-related coagulopathy can complicate the management of these patients, necessitating more vigilant monitoring and potentially different therapeutic approaches [7]. Previous studies have also highlighted the impact of COVID-19 on the cardiovascular system, including myocardial injury and arrhythmias [14]. A trauma cohort showed a higher incidence of cardiac events in the COVID-positive group (3.2% vs. 0.9%, *p* = 0.023) [15].

However, there is limited research on the effect of COVID-19 infection on the prognosis of trauma patients. Previous research results on the difference in prognosis according to the presence of COVID-19 infection in trauma patients are conflicting.

It is known that COVID-19 shows significant differences in morbidity and prognosis depending on how well it is managed from a public health perspective. Therefore, the level of management in a specific country can significantly affect the prognosis of trauma patients with COVID-19 infection. South Korea is known as one of the countries that has best managed the COVID-19 pandemic during the pandemic period. As of 16 November 2023, South Korea reported 693 deaths per million with a total of 35,934 deaths, in stark contrast to countries like the United States (3364 deaths per million), United Kingdom (3426), Brazil (3272), and Italy (3261). These figures reflect South Korea’s success, especially considering that it achieved these results without enforcing strict lockdowns or border closures, unlike many other countries that exhibited higher mortality rates. This outcome resulted from efficient strategies like extensive testing and rigorous contact tracing [16,17,18,19,20,21]. The authors’ hospital established a Task Force team and dedicated 56 beds for COVID-19 response starting from February 2020, and in December 2020, it was designated as a COVID-19 hub hospital by the government. Given these unique circumstances, extrapolating results from other countries’ studies directly to the South Korean context may not be entirely applicable. This study is thus focused on investigating the impact of COVID-19 infection on the prognosis of trauma patients in South Korea, with findings potentially providing insights for countries with similar public health management strategies during the COVID-19 pandemic.

This study explores the association between COVID-19 infection and the outcomes of traumatized patients in South Korea. Our hypothesis was that COVID-19 infection would have an adverse effect on the prognosis of trauma patients. This study will provide valuable information for medical professionals and healthcare providers to better understand the impact of COVID-19 on the management of trauma patients and improve their outcomes.

## 2. Materials and Methods

This retrospective cohort study was conducted on trauma patients admitted to the authors’ regional trauma center and regional emergency medical center over a period of 2 years, from 1 June 2020 to 31 May 2022. Data on these patients were collected from the Korean Trauma Data Bank (KTDB) and electronic medical records. During the study period, a total of 6312 trauma patients were recorded. Among them, a total of 2106 patients were excluded from this study based on the following criteria: individuals under 19 years of age, dead on arrival (D.O.A.) patients, those who returned home without ward admission following emergency room treatment, patients transferred to other hospitals for mild conditions or other reasons, and individuals without trauma as determined by chart review. Consequently, the final analysis was conducted on 4206 patients.

During the specified period, all patients underwent COVID-19 PCR testing upon admission using the PowerChekTM 2019-nCoV Real-time PCR Kit (KogeneBiotech, Seoul, Republic of Korea). The results were reported within 6 to 24 h. In cases where the first test result was equivocal, a retest was conducted, and if the second result was positive, the case was recorded as positive. The enrolled patients were divided into two groups for analysis, those who tested positive and those who tested negative on COVID-19 PCR at the time of admission.

We collected various data including age, sex, injury mechanism, physiological parameters upon emergency room admission (such as body temperature, systolic arterial blood pressure (SBP), heart rate, respiratory rate, and the Glasgow Coma Scale (GCS)), Revised Trauma Score (RTS), Injury Severity Score (ISS), transfusion amount within 24 h, complications (such as pneumonia, acute kidney injury (AKI), sepsis, and wound infection), duration of stay in the intensive care unit, overall hospital stay, and mortality rates. An ISS > 15 was considered to indicate severe trauma [22].

The collected data were analyzed using SPSS/WIN 25.0 (IBM Corp. Armonk, NY, USA), and the specific methods are described as follows: General and clinical characteristics of the subjects were analyzed using descriptive statistics. Clinical outcomes between groups were analyzed using the chi-square test, independent *t*-test, and ANOVA. Factors affecting the mortality rate of trauma patients were analyzed using logistic regression.

The study was conducted after obtaining approval from the Institutional Review Board (IRB) of G University Hospital in I City (IRB No. GAIRB2022-350). As this study is a secondary data analysis using the Korean Trauma Data Bank (KTDB), it was conducted after obtaining approval for exemption from obtaining consent from the study subjects. The data were collected using the KTDB, and all identifying information of the subjects was anonymized using alphabets and numbers. To protect personal information, the collected data were securely stored on the researcher’s personal computer with a password, and the data will be discarded after completion of the study. The data were used for research purposes only.

## 3. Results

### 3.1. Differences in General and Clinical Characteristics between Groups

The total number of subjects in this study was 4206, including 125 in the COVID-19-positive group and 4081 in the COVID-19-negative group. There was no significant difference in age between the two groups, with the positive group having a mean age of 59.35 ± 17.40 years and the negative group having a mean age of 59.56 ± 18.92 years. Both groups had more male than female patients, with no significant difference between them (Table 1).

There were no significant differences between the groups in terms of the admission route, mode of transportation, intentionality, and mechanism of injury.

In the positive group, the RTS was significantly lower (7.3462 ± 0.9842 vs. 7.6450 ± 0.6347, *p* ≤ 0.001), and the total ISS score was significantly higher (15.46 ± 11.86 vs. 11.69 ± 8.91, *p* ≤ 0.001) than in the negative group. The positive group had significantly higher rates of intensive care unit admissions (31.2% vs. 27.4%, *p* ≤ 0.001) and surgical/vascular thrombectomy procedures (23.2% vs. 12.2%, *p* ≤ 0.001) than the negative group. Furthermore, the positive group exhibited a significantly higher blood transfusion rate (26.4% vs. 14.8%, *p* < 0.001). There were no significant differences in mechanical ventilation duration or intensive care unit stay between the groups. However, the total length of hospital stay was significantly longer in the positive group than in the negative group (32.20 ± 32.51 vs. 14.53 ± 16.03, *p* < 0.001). There was no statistically significant difference in in-hospital mortality between the two groups (4% vs. 5.6%, *p* = 0.60) (Table 1). Even though there were statistical differences between groups, the differences in GCS, RTS, and ISS were clinically negligible.

In this study, we conducted a subgroup analysis of severely injured patients with an ISS of 15 or higher and compared them according to their COVID-19 status (Table 2). Among the 1175 severely injured patients, 53 were COVID-19-positive. There were no significant differences in age, sex, or injury mechanism between the groups of severely injured patients. However, in the positive group, the RTS was lower (6.8465 ± 1.2645 vs. 7.2449 ± 1.0459, *p* = 0.008), and ISS was higher (26.26 ± 10.48 vs. 23.64 ± 7.61, *p* = 0.016). There were no significant differences in mechanical ventilation duration or intensive care unit stay, but the total length of hospital stay was significantly longer in the COVID-19-positive group of severely injured patients (53.11 ± 35.84 vs. 23.64 ± 23.36, *p* < 0.001). However, among severely injured patients, there were no differences in intensive care unit admission rates, surgical rates, or blood transfusion rates according to COVID-19 status. Nonetheless, among severely injured patients, those who were COVID-19-positive had a lower mortality rate than those who were COVID-19-negative (5.7% vs. 16.4%, *p* = 0.037) (Table 2).

### 3.2. Differences in Complication Rates between Groups

The types of complications were compared between groups, focusing on major complications that affect the main causes of death and length of stay in the intensive care unit and hospitalization period of trauma patients (Table 3). The rate of pressure sores (8.8% vs. 2.3%, *p* ≤ 0.001), surgical site infections (2.4% vs. 0.8%, *p* = 0.044), pneumonia (8.8% vs. 4.1%, *p* = 0.007), and catheter-related bloodstream infections (1.6% vs. 0.3%, *p* = 0.013) in the positive group were higher than in the negative group.

The pattern of complications in the group of severely injured patients was similar. The incidence rates of pressure ulcers (18.9% vs. 4.5%, *p* < 0.001), pneumonia (18.9% vs. 9.1%, *p* = 0.018), and catheter-related bloodstream infections (3.8% vs. 0.9%, *p* = 0.041) were higher in severely injured patients who were COVID-19-positive than in those who were COVID-19-negative (Table 4).

### 3.3. Factors Influencing the Mortality Rate of the Subjects

The results of logistic regression analysis were statistically significant (χ2 = 486.361, *p* < 0.001), and the explanatory power was 39.2% according to the Nagelkerke R2. The logistic regression analysis showed that COVID-19 infection status, age, GCS, ISS, and transfusion status were significant factors influencing the mortality rate (Table 5).

The odds ratio for the COVID-19-positive group compared to the negative group was 0.29 (95% CI = 0.10–0.84, *p* = 0.022), and the odds ratio increased significantly to 1.04 (95% CI = 1.03–1.06, *p* ≤ 0.001) with increasing age. In addition, the odds ratio decreased to 0.75 (95% CI = 0.71–0.78, *p* ≤ 0.001) with decreasing GCS, and the odds ratio increased significantly to 1.06 (95% CI = 1.04–1.08, *p* ≤ 0.001) with increasing ISS score.

## 4. Discussion

This study was designed to investigate the impact of COVID-19 infection on trauma patients during the COVID-19 pandemic period, in a situation where the spread of COVID-19 was relatively well-controlled. The results of this study showed different outcomes from our initial hypothesis. While COVID-19-positive patients had a worse prognosis than negative patients in terms of clinical indicators such as pressure sores, surgical site infections, pneumonia, and catheter-related bloodstream infections, there was no significant difference in mortality between the COVID-19 positive and negative groups. Moreover, the mortality of the COVID-19 negative group was marginally significantly higher than that of the COVID-19 positive group in severely traumatized patients. This significant difference may have been, by chance, caused by the small sample size of COVID-19 (+) patients. Nevertheless, the fact that the COVID-19 positive group did not show a higher mortality rate is a result that contradicts previous research findings. Additionally, in support of that, the regression analysis in this study reveals that COVID-19 positivity was not a significant factor, aligning with our primary research objective. While age and Injury Severity Score (ISS) showed statistical significance, they did not translate into substantial clinical differences. Conversely, the volume of blood transfusions and Glasgow Coma Scale (GCS) scores were found to be significant both statistically and clinically. These results suggest that COVID-19 status did not significantly alter the clinical outcomes of trauma patients, while factors such as the need for blood transfusions and lower GCS scores play a more critical role in determining patient prognosis. Although there are not many studies investigating the impact of COVID-19 infection on the prognosis of trauma patients, the existing research results have consistently demonstrated that COVID-19-positive patients had a worse prognosis in terms of mortality compared to those who were not infected with COVID-19 [7,23,24,25].

A retrospective study conducted in Iran compared COVID-19 PCR-positive and negative trauma patients. The clinical data of 100 patients in each group from a single trauma center were analyzed. The COVID-19 PCR-positive group did not show any significant differences in comorbidities compared to the PCR-negative group. However, multivariate analysis revealed that COVID-19 PCR positivity was associated with a longer intensive care unit (ICU) stay (odds ratio = 1.135, 95% CI = 1.073–1.200, *p* < 0.001) and higher mortality (odds ratio = 2.884, 95% CI = 1.122–7.414, *p* = 0.028). The authors suggest that the differences in morbidity and mortality may be due to varying severity of COVID-19 or the use of steroids in the PCR-positive group [26].

In Scotland, a study targeting mainly orthopedic trauma patients found that COVID-19 nearly doubled the postoperative mortality risk and resulted in a lower survival rate. The study identified older age, increasing morbidity, and certain types of fractures as risk factors for developing COVID-19 postoperatively. Consequently, the authors recommended that surgery should be postponed until the resolution of COVID-19 [23].

Another study conducted in California, United States, investigated the impact of COVID-19 on trauma patients in Level-I and II trauma centers. The study included 20,448 trauma patients and utilized a 1:2 propensity score model to match 53 COVID-19-positive patients with 106 non-COVID-19 patients. The study found that COVID-19-positive patients had higher mortality rates (9.4% vs. 1.9%, *p* = 0.029), higher rates of pneumonia, longer mean length of stay, and longer ICU stays than non-COVID-19 patients [8]. In our study, complications such as pneumonia, pressure ulcers, catheter-related bloodstream infections, and surgical site infections were notably higher in the COVID-19-positive group. According to the literature, it appears that trauma patients, regardless of the severity of their condition, are negatively affected by COVID-19 infection. There are several hypotheses regarding the reasons for poor outcomes in COVID-19-positive trauma patients demonstrated in this study. First, some hypothesize that it is related to the stresses of surgery and mechanical ventilation in the setting of concurrent infection [11,27]. Second, others suggest that it is due to systemic inflammatory response syndrome (SIRS) and multi-organ dysfunction syndrome (MODS) caused by COVID-19 [28,29]. Finally, some suggest that it is due to the effects of steroids used to treat COVID-19 patients [30,31,32]. Although this study was not designed to explain these poor outcomes in COVID-19-positive trauma, the authors believe that the aforementioned causes may have acted in combination.

Although the studies discussed above were designed differently and conducted in different environments, meaning that they cannot be simply compared, the impact of COVID-19 infection may have been influenced by the public health situations in each country. Iran showed a relatively large difference with an odds ratio of 2.88, Scotland had an odds ratio of 1.89, and the United States showed a mortality rate of 9.4% in COVID-19-positive patients, which was more than 4 times higher than the 1.9% in negative patients [8,23,26].

Our research findings indicate that COVID-19 positivity had a negative impact on several clinical factors. However, it is difficult to explain the discrepancy in mortality compared to previous studies. In the study conducted by Sozzi et al., it was reported that the group of patients who were asymptomatic but tested positive for COVID-19 experienced a higher rate of complications and longer hospital stays, yet the mortality rate did not significantly differ from that of the COVID-19 negative patient group [15]. Similarly, Klutts et al. noted an increase in the length of hospital stays and ventilator days for asymptomatic COVID-19 positive patients; however, they stated that the mortality rate was too low to make a meaningful comparison [33]. This could potentially be due to differences in the public health environment between countries during the COVID-19 pandemic period, where there were variations in the incidence of COVID-19 patients, rate of vaccination, and coping strategies between countries. Additionally, another possible factor to consider as a cause of these differing results is that the proportion of COVID-19-positive patients in our study was substantially lower than those of previous studies. As a result, there is a possibility that these differing results may reflect statistical coincidences.

The authors acknowledge that this study has several limitations. Firstly, it was a retrospective study that inherently had limitations in terms of data collection and analysis. Secondly, the proportion of COVID-19 patients in the study population was relatively small, which may have led to statistical coincidences and could be attributed to the low incidence of COVID-19 in South Korea. The control of variables within the study also raise questions. While the research accounts for a range of clinical variables, it remains unclear whether all potential confounding factors, such as patients’ pre-existing health conditions, lifestyle choices, and socio-economic backgrounds, were adequately controlled. These factors could significantly influence the study’s outcomes. Another limitation arises from the regional context of the study. Conducted in South Korea, the findings are deeply rooted in the country’s specific public health and healthcare system. This context may not be directly applicable to other countries or populations with different healthcare systems and cultural backgrounds, thus limiting the global applicability of the results. The statistical analysis of the study, while indicating certain significant differences, does not clearly establish the clinical relevance of these findings. For instance, the reported statistical significance in variables like GCS, RTS, and ISS does not necessarily translate into clinical significance, a distinction that appears to be underexplored in the study. The interpretation of the mortality outcomes, particularly the lack of significant differences between COVID-19 positive and negative groups, is intriguing. It suggests a potentially limited impact of COVID-19 on mortality rates. However, this aspect warrants further investigation, especially concerning other clinical outcomes and the long-term effects of the infection. Finally, only COVID-19 positivity was investigated, and other factors such as viral load, immunological markers, and medications were not measured.

These limitations highlight the need for careful interpretation of the study’s findings and suggest areas for improvement in future research endeavors.

## 5. Conclusions

In conclusion, our study highlights the necessity for heightened monitoring and management of specific complications in COVID-19 positive trauma patients. These include increased rates of pressure sores, surgical site infections, pneumonia, and catheter-related bloodstream infections. Despite these increased complication rates, our findings indicate a lower mortality rate in COVID-19 positive trauma patients. This paradox warrants further investigation to understand the underlying reasons, potentially involving the role of different public health strategies and medical interventions. Future research should focus on unraveling these complexities to improve the management and outcomes of trauma patients during the ongoing pandemic.

## Figures and Tables

**Table 1:** Comparison of demographic and clinical characteristics between the COVID-19-positive (COVID-19) group and the COVID-19-negative (non-COVID-19) group (N = 4206).

Variable	Categories	n (%), M ± SD	COVID-19 (n = 125)	Non-COVID-19 (n = 4081)	*p*
			n (%), M ± SD	n (%), M ± SD	
Age (years)		59.5 ± 18.8	59.3 ± 17.4	59.5 ± 18.9	0.904
Sex (male)	Male	2645 (62.9)	83 (66.4)	2562 (62.8)	0.409
Injury mechanism	Driver TA	388 (9.2)	10 (8.0)	378 (9.3)	0.292
	Bike	118 (2.8)	2 (1.6)	116 (2.8)	
	Motorcycle	330 (7.8)	13 (10.4)	317 (7.8)	
	Pedestrian TA	382 (9.2)	17 (13.6)	365 (8.9)	
	Fall	835 (19.9)	27 (21.6)	808 (19.8)	
	Slip	1440 (34.2)	38 (30.4)	1402 (34.4)	
	Struck	265 (6.3)	7 (5.6)	258 (6.3)	
	Penetrating	198 (4.7)	6 (4.8)	192 (4.7)	
	Machine	47 (1.1)	0 (0.0)	47 (1.2)	
	Burn	10 (0.2)	0 (0.0)	10 (0.2)	
	Other	106 (2.5)	0 (0.0)	106 (2.6)	
	Unknown	87 (2.1)	5 (4.0)	82 (2.0)	
ER outcome	Send to ward	2520 (59.9)	57 (45.6)	2463 (60.4)	<0.001
	Send to ICU	1158 (27.5)	39 (31.2)	1119 (27.4)	
	Send to OR/IR	528 (12.6)	29 (23.2)	499 (12.2)	
Vital signs	SBP	144.9 ± 31.7	139.9 ± 36.5	145.1 ± 31.5	0.073
	PR	87.1 ± 18.5	90.2 ± 19.6	87.0 ± 18.5	0.059
	RR	19.7 ± 3.6	20.3 ± 3.6	19.7 ± 3.6	0.078
	BT	36.6 ± 0.6	36.4 ± 0.7	36.6 ± 0.6	0.007
GCS		14.2 ± 2.3	13.4 ± 3.3	14.2 ± 2.3	<0.001
Transfusion		638 (15.2)	33 (26.4)	605 (14.8)	<0.001
Ventilator (days)		7.1 ± 12.7	9.0 ± 6.4	7.0 ± 12.9	0.550
ICU (days)		6.5 ± 11.3	8.5 ± 11.2	6.4 ± 11.3	0.157
RTS		7.6 ± 0.6	7.3 ± 0.9	7.6 ± 0.6	<0.001
ISS		11.8 ± 9.0	15.4 ± 11.8	11.6 ± 8.9	<0.001
LOH (days)		15.0 ± 17.0	32.2 ± 32.5	14.5 ± 16.0	<0.001
Mortality		234(5.6)	5 (4.0)	229 (5.6)	0.439

TA: traffic accident; ER, emergency room; ICU, intensive care unit; OR, operation room; IR, intervention room; SBP, systolic blood pressure; PR, pulse rate; RR, respiratory rate; BT, body temperature; GCS; Glasgow Coma Scale; RTS, Revised Trauma Score; ISS, Injury Severity Score; LOH, length of hospital stay.

**Table 2:** Comparison of demographic and clinical characteristics between the COVID-19-positive (COVID-19) subgroup and the COVID-19-negative (non-COVID-19) subgroup in severe trauma patients. (N = 1175).

Variable	Categories	n (%), M ± SD	COVID-19 Subgroup (n = 53)	Non-COVID-19 Subgroup (n = 1122)	*p*
			n (%), M ± SD	n (%), M ± SD	
Age (years)		56.7 ± 17.7	54.4 ± 15.5	56.9 ± 17.8	0.322
Sex	Male	893 (76.0)	40 (75.5)	853 (76.0)	0.927
Injury mechanism	Driver TA	134 (11.4)	4 (7.5)	130 (11.6)	0.612
	Bike	37 (3.1)	1 (1.9)	36 (3.2)	
	Motorcycle	136 (11.6)	8 (15.1)	128 (11.4)	
	Pedestrian TA	181 (15.4)	10 (18.9)	171 (15.2)	
	Fall	322 (27.4)	17 (32.1)	305 (27.2)	
	Slip	214 (18.2)	4 (7.5)	210 (18.7)	
	Struck	73 (6.2)	4 (7.5)	69 (6.1)	
	Penetrating	20 (1.7)	1 (1.9)	19 (1.7)	
	Machine	13 (1.1)	0 (0)	13 (1.2)	
	Burn	1 (0.1)	0 (0)	1 (0.1)	
	Other	1 (0.1)	0 (0)	1 (0.1)	
	Unknown	43 (3.7)	4 (7.5)	39 (3.5)	
ER outcome	Send to ward	226 (19.2)	5 (9.4)	221 (19.7)	0.097
	Send to ICU	658 (56.0)	30 (56.6)	628 (56.0)	
	Send to OR/IR	291 (24.8)	18 (34.0)	273 (24.3)	
Vital signs	SBP	138.6 ± 37.3	135.2 ± 44.1	138.8 ± 37.0	0.498
	PR	91.4 ± 22.5	91.4 ± 21.8	91.4 ± 22.5	0.996
	RR	21.0 ± 4.8	20.4 ± 4.2	21.0 ± 4.9	0.317
	BT	36.4 ± 0.8	36.1 ± 0.9	36.4 ± 0.8	0.019
GCS		12.7 ± 3.7	11.7 ± 4.3	12.7 ± 3.7	0.065
Transfusion		426 (36.3)	24 (45.3)	402 (35.8)	0.162
Mechanical ventilator (days)		4.4 ± 4.6	9.6 ± 6.2	7.5 ± 14.0	0.571
ICU (days)		8.6 ± 13.7	11.6 ± 12.5	8.5 ± 13.8	0.165
RTS		7.2 ± 1.0	6.8 ± 1.2	7.2 ± 1.0	0.008
ISS		23.7 ± 7.7	26.2 ± 10.4	23.6 ± 7.6	0.016
LOH (days)		24.9 ± 24.8	53.1 ± 35.8	23.6 ± 23.3	<0.001
Mortality		187 (15.9)	3 (5.7)	184 (16.4)	0.037

TA: traffic accident; ER, emergency room; ICU, intensive care unit; OR, operation room; IR, intervention room; SBP, systolic blood pressure; PR, pulse rate; RR, respiratory rate; BT, body temperature; GCS; Glasgow Coma Scale; RTS, Revised Trauma Score; ISS, Injury Severity Score; LOH, length of hospital stay.

**Table 3:** Comparison of outcomes between the COVID-19-positive (COVID-19) group and the COVID-19-negative (non-COVID-19) group (N = 4206).

Variable	Categories	n (%)	COVID-19 (n = 125)	Non-COVID-19 (n = 4081)	*p*
			n (%)	n (%)	
Complication	ARF	45 (1.1)	0 (0.0)	45 (1.1)	0.238
	ARDS	6 (0.1)	0 (0.0)	6 (0.1)	0.668
	Sore	106 (2.5)	11 (8.8)	95 (2.3)	<0.001
	SSI	34 (0.8)	3 (2.4)	31 (0.8)	0.044
	DVT	68 (1.6)	3 (2.4)	65 (1.6)	0.481
	Pneumonia	177 (4.2)	11 (8.8)	166 (4.1)	0.009
	PTE	22 (0.5)	2 (1.6)	20 (0.5)	0.090
	UTI	74 (1.8)	4 (3.2)	70 (1.7)	0.214
	CRABSI	14 (0.3)	2 (1.6)	12 (0.3)	0.013
	Severe sepsis	25 (0.6)	0 (0.0)	25 (0.6)	0.380

ARF, acute renal failure; ARDS, adult respiratory distress syndrome; SSI, surgical site infection; DVT, deep venous thrombosis; PTE, pulmonary thromboembolism; UTI, urinary tract infection; CRABSI, catheter-related bloodstream infection.

**Table 4:** Comparison of outcomes between the COVID-19-positive (COVID-19) subgroup and the COVID-19-negative (non-COVID-19) subgroup in severe trauma patients (N = 1175).

Variable	Categories	n (%)	COVID-19 Subgroup (n = 53)	Non-COVID-19 Subgroup (n = 1122)	*p*
			n (%)	n (%)	
Complication	Overall	240 (100.0)	16 (30.2)	224 (20.1)	0.077
	ARF	24 (2.0)	0 (0.0)	24 (2.1)	0.282
	ARDS	2 (0.2)	0 (0.0)	2 (0.2)	0.758
	Sore	60 (5.1)	10 (18.9)	50 (4.5)	<0.001
	SSI	19 (1.6)	1 (1.9)	18 (1.6)	0.873
	DVT	37 (3.1)	2 (3.8)	35 (3.1)	0.790
	Pneumonia	112 (9.5)	10 (18.9)	102 (9.1)	0.018
	PTE	16 (1.4)	2 (3.8)	14 (1.2)	0.121
	UTI	42 (3.6)	3 (5.7)	39 (3.5)	0.403
	CRABSI	12 (1.0)	2 (3.8)	10 (0.9)	0.041
	Severe sepsis	10 (0.9)	0 (0)	10 (0.9)	0.490

ARF, acute renal failure; ARDS, adult respiratory distress syndrome; SSI, surgical site infection; DVT, deep venous thrombosis; PTE, pulmonary thromboembolism; UTI, urinary tract infection; CRABSI, catheter-related bloodstream infection.

**Table 5:** Results of logistic regression analysis to identify factors influencing mortality in trauma patients (N = 4206).

Variable	B	S.E.	*p*	Odd Ratio	95% Confidence Interval
COVID-19	−1.23	0.53	0.022	0.29	0.10–0.84
Age	0.04	0.06	<0.001	1.04	1.03–1.06
GCS	−0.30	0.02	<0.001	0.75	0.71–0.78
ISS	0.06	0.01	<0.001	1.06	1.04–1.08
Transfusion	1.42	0.21	<0.001	4.12	2.74–6.19

GCS, Glasgow Coma Scale; ISS, Injury Severity Score.

## Data Availability

The data presented in this study are available on request from the corresponding author.
